# Characterization of Hydrochloride and Tannate Salts of Diphenhydramine

**DOI:** 10.4103/0250-474X.44598

**Published:** 2008

**Authors:** T. D. Nandgude, K. S. Bhise, V. B. Gupta

**Affiliations:** Department of Pharmaceutics, B. R. Nahata College of Pharmacy, Mhow-Neemuch Road, Mandsaur- 458 001, India; 1 Department of Pharmaceutics, M. C. E. Society’s, Allana College of Pharmacy, Camp, Pune-411 001, India

**Keywords:** Diphenhydramine hydrochloride, tannate, solubility, stability

## Abstract

Proper characterization is an important aspect of any dosage form design. The objective of this work was to characterize tannate salt and hydrochloride salt of diphenhydramine. As a part of characterization studies, Differential scanning calorimetry was used to investigate thermal effects and nature of salts, supported by X-ray powder diffraction. Scanning electron microphotographs was used to surface topography of salts of diphenhydramine. Fourier-transform infrared spectroscopy, solubility study and flowability studies were carried out as part of characterization. Differential scanning calorimetry and X-ray powder diffraction studies indicated amorphous nature of the tannate while hydrochloride salt has crystalline properties. Scanning electron microphotographs indicated the differences in surface topography between both the salts. Solubility studies at different pH showed pH dependant solubility of both the salts and less solubility of tannate. Stability of bulk drug at accelerated conditions of 40^°^/75% RH was determined for both salts. Good stability of both salts was observed.

The chemical, biological, physical and economical characteristics of medical agent can be manipulated and hence, often optimized by conversion to a salt form. Choosing the appropriate salt, however, can be a very difficult task, since each salt imparts unique properties to the parent compound^1^. For the past five decades scientific data collectively known as characterization or preformulation studies have been developed for supporting the dosage form design of a new drug and its quality control^1^,^2^. The salt form is known to influence a number of physicochemical properties of the parent compound. Ideally, it would be desirable if one could predict how a pharmaceutical agent’s properties would be affected by salt formation^1^,^3^.

Diphenhydramine (DPH) belongs to the class of ethanolamine H_1_ receptor antagonist and possesses in addition to antihistaminic activity, a significant anticholinergic effect^4^. DPH, water insoluble drug, is administered in the form of its acid salt such as hydrochloride (HCl) to improve water solubility, but are disadvantageous due to fast absorption in the mammalian body^5^.

In the present study, two solid-state salts of DPH were characterized for thermal study by differential scanning calorimetry (DSC), crystallographic study using X-ray powder diffraction (X-RPD), microscopic study by scanning electron microphotographs (SEM) and spectroscopic characteristics study by Fourier-transform infrared spectroscopy (FT-IR). Influence of morphology of salts on flow behavior was also investigated. These salts were also studied for pH dependent solubility and accelerated stability studies.

## MATERIALS AND METHODS

DPH HCl was generously gifted by Kamud Drugs Pvt. Ltd., Sangli, India. DPH tannate obtained as gift sample from Scitech Healthcare Pvt. Ltd., Mumbai. All other chemicals and reagents used were of analytical grades.

### Determination of λmax^6^:

A solution 0.127% w/v of DPH HCl and DPH tannate were prepared. Accurately weighed (Digital Balance, Model-BL-220 II, Contech Ltd., Mumbai, India) 127 mg of drug dissolved in 100 ml methanol and filtered through a 0.2 μ membrane filter. A filtered solution was kept in fused silica cell and UV spectrum (Model-V-530, Jasco Pharmaspec, Tokyo, Japan) was recorded in the wavelength range 200-800 nm.

### Infrared spectroscopy^6^,^7^:

Fourier-transform infrared (Model-4100, Jasco Corporation, Tokyo, Japan) spectra of DPH HCl and DPH tannate were obtained over the wave number range of 4000 to 400 cm^-1^.

### Surface Topography^8^:

The surface topography of pure drug was analyzed with a SE Microscope (Model-SU-SEM-Probe, Camecha, France) operated at an accelerated voltage of 25 kv and at magnifications 1000, 2000 and 4000, respectively.

### Flowability^9^,^10^:

Angle of repose was determined by the fixed funnel method. Accurately weighed powder blend was poured in the glass funnel. The height of funnel was adjusted in such a way that the tip of the funnel just touched the apex of the heap of powder. The powders were allowed to flow through a glass funnel freely onto a clean surface. The diameter of the powder cone so formed was measured and angle of repose, Tan Ø= h/r, Therefore; θ= tan^-1^ (h/r). Tapping cylinder method was used for determining bulk density, ρ_b_ = Weight of the powder (W)/Volume of the packing (V_b_) and tapped density, ρ_t_ =Weight of the powder (W)/Tapped volume of the packing (V_t_), using Bulk Density Apparatus (Elico, India). Powder was taken in a 50 ml measuring cylinder and the initial volume (bulk volume) and the volumes after 50 tapping were measured. Carr’s Compressibility Index^11^ (%) = [(ρ_t_ -ρ_b_)×100]/ρ_t_ and Haussner’s ratio = ρ_t_/ρ_b_ were calculated.

### X-ray powder diffraction^6^,^12^:

The X–RPD patterns of pure drugs were recorded (X- ray diffractometer, Model-1729, Philips PW). Samples were irradiated with monochromatized Cu Ka radiation (λ (Cu) = 1.542 A°) and analyzed between 2° and 60° 2θ. The voltage and current used were 30 kv and Ma, respectively.

### Differential scanning calorimetry^6^,^12^,^13^:

DSC thermograms were obtained (Model-9900, Du-Pont, USA), the samples were exposed to heating rate of 10°/min over a temperature range of 10–250° under nitrogen purging. Both the melting point and the heat of fusion are obtained from DSC; the peak area is equal to the heat of fusion, H_f,_ in units of calories per gram. The change in enthalphy, that is enthalpy of fusion, ΔH, is equal to the difference between the heat flow to or from the sample, Qs, and the heat flow to or from the reference, Qr, that is ΔH = Qs – Qr.

### Solution properties^14^,^15^:

One percent solutions of pure drugs prepared by using water as a solvent and pH of freshly prepared solutions were determined by pH meter (Toshniwal Instruments Pvt. Ltd., Ajmer, India). To find out saturation solubility and pH dependent solubility, saturated solutions were prepared by dissolving excess amounts of DPH HCL and DPH tannate in to water, methanol, ethanol 95% and phosphate buffer solutions with pH ranging from 1.2 to 8.0, which covers the normal pH range of the human gastrointestinal tract, at room temperature (25 to 27°). The pH of the solution was adjusted by hydrochloric acid and sodium hydroxide. A suspension was shaken by Griffin Flask Shaker (Wrist Action Shaking Machine) for 6 h (24 h when the solubility was < 0.1 mg/ml). Small volumes of these solutions were filtered through a 0.2 μ membrane filter and diluted as necessary, then kept in fused silica cell and absorbance determined spectrophotometricaly using an UV/Vis Spectrophotometer at 258 nm and 276 nm, respectively.

### Accelerated stability studies^15^–^17^:

To assess the solid - state stability, studies were done at 40°/75% RH for three months; incubator (Lab Tech Instruments, Kasliwal Brothers, Indore, India) was used to maintain the temperature. Humidity chamber of 75% RH was prepared using NaCl. Saturated salt solutions remained in contact with excess solid salt in selected desiccators. The chamber was equilibrated at room temperature for at least 24 hours before use. Analysis was carried out by FT-IR and observed organoleptic properties.

## RESULTS AND DISCUSSION

**Fig. 1:**
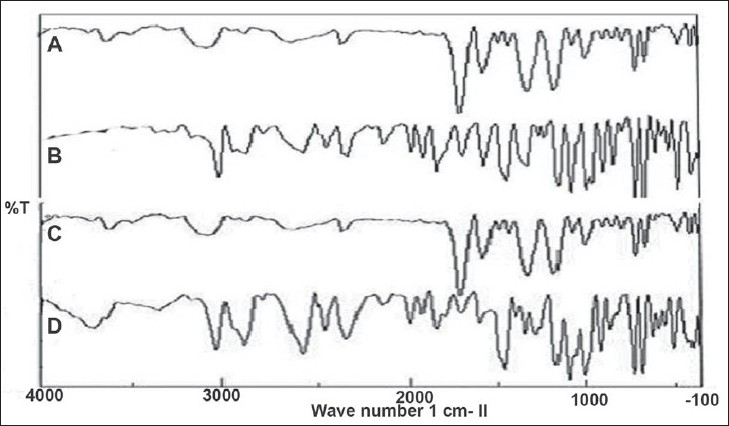
FT-IR spectra of DPH HCL and DPH tannate salts FT-IR spectra of (A) DPH tannate, (B) DPH HCl, (C) DPH tannate after stability and (D) DPH HCl after stability

Primary importance in the development of oral dosage forms is taste acceptability. Since taste is a chemical sense, a tannate salt of DPH is tasteless and HCl salt of DPH is highly bitter in taste. The wavelength of maximum absorbance (λmax) in methanol solvent was found to be 258 nm and 276 nm for HCl salt and tannate salt, respectively. IR spectra of DPH HCl (fig. 1) indicated characteristic peaks belonging to major functional groups which are similar to standard peaks, such as aromatic CH stretch (3035.41 cm^-1^), -N (CH_3_)_2_ HCl (2400-2700 cm^-1^), aromatic rings skeletal vibrations (1592.91,1407 cm^-1^), CH_2_ bending (1461.78 cm^-1^), CH_3_ bending (1342.21 cm^-1^), C-O-C stretching (1106.94 cm^-1^) and C-H out of plane deformation of mono-substituted phenyl (717, 760 cm^-1^). DPH tannate (fig. 1) with these characteristic peaks indicated O-H bonding (1600 cm^-1^), C=O stretching (1735 cm^-1^).

**Fig. 2:**
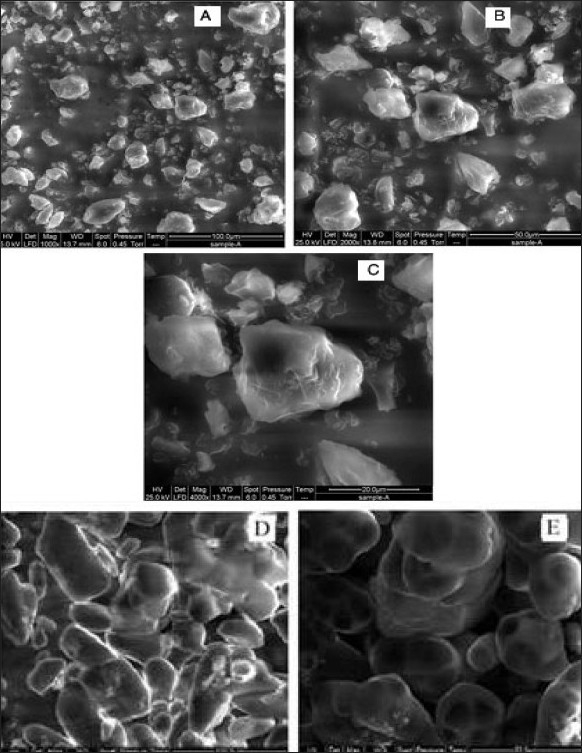
SEMicrophotographs of DPH salts at different magnifications SEMicrophotographs of DPH salts at different magnifications, (A) DPH HCL at 1000 X, (B) at 2000 X and (C) at 4000X; (D) DPH tannate at 1000 X and (E) at 2000 X

Particle morphology influences various pharmaceutical engineering and biopharmaceutical parameters such as flowability characteristics of drug powder. SEM of the DPH HCl showed (fig. 2) crystalline salt is in the form of irregular in shape having cracks on surface with pores and has rough surface. SEM of DPH tannate (fig. 2) was regular in particle shape and size and has smooth surface as well as absence of pinholes on surface.

**Fig. 3:**
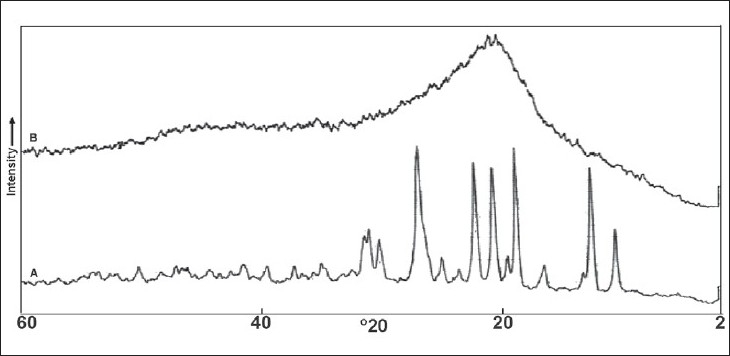
X-RPD patterns of DPH Salts X-RPD patterns of (A) DPH HCl and (B) DPH tannate

Values for angle of repose indicated good flow properties of salts and this was further supported by lower Carr’s compressibility index values and Haussner’s ratio (Table 1). X-RPD patterns (fig. 3) of DPH tannate are quite distinct from those produced by the DPH HCl, which showed significant decrease in the intensity of the peaks in the region of lower and higher 2θ values (up to 10 and above 28°), where as peaks between the region 2θ value 10 to 28° have broadened peaks with high intensity. The maximum intensity peak i.e. I_O_ has been shifted from 2θ values 24.8 to 26.9°. X-RPD patterns may be attributed to this wide variety crystalline structure of DPH HCl and amorphous nature of DPH tannate.

**TABLE 1:** FLOW PROPERTIES OF DPH SALTS

DPH Salts	Bulk density (g/ml)	Tapped density (g/ml)	Carr’s compressibility Index (%)	Haussener’s ratio	Angle of repose (θ)
HCl	0.38±0.01	0.45±0.02	15.55±0.05	1.18±0.02	39.09±0.02
Tannate	0.34±0.01	0.41±0.03	17.07±0.01	1.20±0.02	30.06±0.06

* All values represent mean±SD (n = 3)

**Fig. 4:**
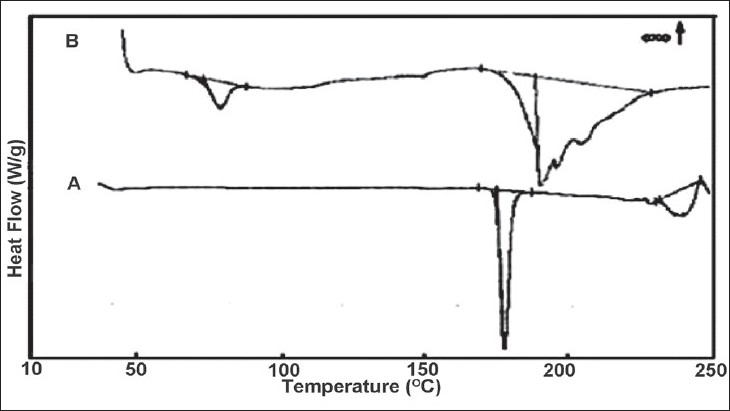
DSC thermograms of DPH salts at a heating rate of 10°/min DSC thermograms of (A) DPH HCl and (B) DPH tannate at a heating Rate of 10°/min

DSC thermograms (fig. 4) of DPH HCl (A) showed broad and asymmetric melting endotherm within 170.58 to 180°. DPH tannate (B) showed glass transition (Tg) peak at 77.05° and relatively very broad and asymmetric melting endotherm between 171 to 227°. The onset and endset temperatures and enthalpy of melting for both drugs are given (Table 2). All thermal data also supported amorphous nature of DPH tannate. The melting point was also determined by capillary method (Table 2), melting points of DPH HCl and DPH tannate were found to be in the range of 169 to 173° and 187 to 193°, respectively.

**TABLE 2:** CHARACTERISTICS OF MELTING BY DSC AND MELTING POINTS

DPH Salts	Onset temp. °	Peak temp. °	Endset temp. °	Enthalpy of fusion (j/g)	Melting Point
HCl	170.58	173.02	180	106	169-171°
Tannate	171	190.20	227	131	185-193°

The onset and endset temperatures and enthalpy of melting by DSC and melting points determined by capillary method of both HCL and tannate salts of DPH

The pH of 1% solution was within 6 to 6.5 and 6 to 6.2, of DPH HCL and DPH tannate, respectively. The pH saturation solubility profiles for DPH HCl and DPH tannate were showed significantly higher solubility of DPH HCl than DPH tannate at all the determined pH values and in water and organic solvents (Table 3). Both, drugs exhibited high solubility at a pH < 2.0. For example, at room temperature, pH 1.2, the solubility of DPH HCl and DPH tannate were 107 and 34 mg/ml, respectively. However, the solubility of DPH HCl, being a weak base, demonstrated a parabolic relationship showing minimum solubility around pH 3.0 to 6.0 and high solubility in both the low acid and alkaline range, appears to dissolve maximally around pH ≤2.0. IR spectra of DPH salts (fig. 1) after stability study did not show any significant changes in the characteristic peaks of DPH tannate, thus indicating good stability of drug at accelerated conditions and DPH HCl shows extra peak at 3600 to 3700 cm^-1^ indicating presence of moisture, but no other significant changes observed. Also no changes were observed in organoleptic properties after stability studies.

**TABLE 3:** SOLUBILITY OF THE DPH SALTS

Solvent	Solubility of salts (mg/ml)		Observations during solubility experiments	
		
	DPH HCl	DPH Tannate	DPH HCl	DPH Tannate
Water	683	13	Freely Soluble	Soluble
pH 1.2 buffer	107	34	Soluble	Soluble
pH 1.8 buffer	96	21	Soluble	Slightly soluble
pH 2.2 buffer	92	-	Soluble	Form brown sticky material
pH 3.0 buffer	84	-	Soluble	Form brown sticky material
pH 3.6 buffer	86	-	Soluble	Form brown sticky material
pH 4.0 buffer	81	-	Soluble	Form brown sticky material
pH 5.5 buffer	84	-	Soluble	Form brown sticky material
pH 6.8 buffer	92	19	Soluble	Slightly soluble
pH 7.4 buffer	98	24	Soluble	Slightly soluble
Methanol	127	104	Freely Soluble	Freely Soluble
Ethanol 95%	88	-	Soluble	Form brown sticky material

A well designed salt selection and optimization study provides a sound base on which to build a rapid and economical product development program. Salt selection involved multiple functions; there is no one size fits all approach. Hence, from the above studies it can be concluded that tannate as well as HCl salts showed good micromeritic properties. Tannate, amorphous, salt showed very less solubility than HCl, crystalline, salt at overall pH range. Tannate salt showed good stability at accelerated conditions. Results shown the feasibility of using tannate salt for formulating simple sustained-release dosage forms.
